# Delayed Supplementation Strategy of Extracellular Vesicles from Adipose-Derived Mesenchymal Stromal Cells with Improved Proregenerative Efficiency in a Fat Transplantation Model

**DOI:** 10.1155/2022/2799844

**Published:** 2022-09-07

**Authors:** Shan Mou, Yuan Li, Di Sun, Muran Zhou, Jialun Li, Lifeng Chen, Shaokai Liu, Jie Yang, Peng Xiao, Jing Tong, Zhenxing Wang, Jiaming Sun

**Affiliations:** ^1^Department of Plastic Surgery, Union Hospital, Tongji Medical College, Huazhong University of Science and Technology, Wuhan 430022, China; ^2^Wuhan Clinical Research Center for Superficial Organ Reconstruction, Wuhan 430022, China

## Abstract

**Background:**

Mesenchymal stromal cells (MSCs) and their secreted extracellular vesicles (MSC-EVs) possess similar proregenerative effects when injected into defects immediately following trauma. However, MSC-EVs are superior to MSCs in terms of storage and rejection reflection, while immediate administration of MSC-EVs is related to several target cells for most donor cells die within few weeks. Besides, the inflammatory cascade is incited, providing an unfavorable environment for target cells. We hypothesized that delayed injection of MSC-EVs might have priority on tissue regeneration than instant injection.

**Method:**

Extracellular vesicles isolated from adipose-derived mesenchymal stromal cells (ADSC-EVs) were administered into human umbilical vein endothelial cells (HUVECs) *in vitro* at different doses. The migration of HUVECs was assessed using the scratch wound healing assay, whereas the length of tubes and number of vessel-like structures formed by HUVECs were determined using tube formation assay. Next, 24 BALB/c nude mice were randomly divided into three groups (*n* = 8). For the EV-delayed group, ADSC-EVs were injected into transplanted fat a week later than the EV-immediate group. The volume and weight of grafts were measured at 3 months after fat transplantation. Further, the number of CD31-possitive vessels and CD206-possitive cells in the fat grafts was quantified.

**Results:**

Compared with the EV-immediate group, the EV-delayed group had a higher fat tissue retention volume (0.11 ± 0.02 mL versus 0.08 ± 0.01 mL), more neovessels (31.00 ± 4.60 versus 24.20 ± 3.97), and fewer cysts. Furthermore, there were more Ki67-positive cells (25.40 ± 7.14 versus 16.20 ± 4.17) and CD206-positive M2 macrophages cells (23.60 ± 3.44 versus 14.00 ± 3.85) in the EV-delayed group than in the EV-immediate group.

**Conclusion:**

Delayed injection of ADSC-EVs promotes fat graft volume retention by stimulating angiogenesis. These findings suggest that delayed supplementation might be a more effective strategy for the application of MSC-EVs in tissue regeneration.

## 1. Introduction

In regenerative medicine, secretome derivatives of mesenchymal stem cell (MSC) such as extracellular vesicles (EVs) are regarded as cell-free therapeutic alternatives to classical MSC therapies [1, 2]. EVs derived from MSC (MSC-EVs) are nanosized vesicles with lipid bilayer outer membranes, mainly carrying microRNAs, proteins and lipids [3]. They play a vital role in intercellular communication [4]. EVs confer therapeutic effects against myocardial infarction, renal injury, liver injury, and second-degree skin burn wounds either through direct external interactions with target cells or via different mechanisms when internalized (receptor­dependent endocytosis, micropinocytosis, plasma membrane fusion, or phagocytosis) [5–12].

MSC-EVs confer proangiogenesis, antifibrosis, and anti-inflammatory effects in different diseases [13–15]. EVs isolated from adipose-derived mesenchymal stromal cells (ADSC-EVs) have been found to promote angiogenesis by delivering miRNA-31 into the affected tissues [16]. The proregenerative functions of MSC-EVs depend on MSC physiological conditions, such as age, senescence, and activation [17]. Numerous animal models have been developed to study the immunomodulating effects of MSC-EVs. Findings from such studies indicate that MSC-EVs may modify the expression of anti-inflammatory and proinflammatory cytokines [18]. In mice with hypoxia-induced pulmonary hypertension, MSC-EVs induced anti-inflammation by inhibiting signal transducer and activator of transcription 3 (STAT3) signaling [19]. Moreover, this cell-free alternative therapy is more immune tolerable compared with MSC therapies. It was reported that intravenous injection of xenogeneic human cardiosphere-derived EVs improved behavioral function in small-clot embolized rabbits [20]. Allogeneic and xenogeneic EVs were reported to promote skin wound healing [21]. Furthermore, EV-based therapies are reproducible, scalable, and controllable, suitable for medical applications [22].

Local injection/transplantation of MSCs has been widely applied to treat many diseases such as acute myocardial infarction, liver injury, and bone defects [23–25]. In most studies, MSCs/MSC-EVs are transplanted into the tissues immediately after injury or disease occurrence [26–28]. Besides, a recent study on merino sheep with a tibial defect found that late transplantation of MSCs triggered bone regeneration [29]. Ideally, inflammation at the site of injury limits oxygen and nutrient supply, thereby impairing survival and subsequent regeneration of MSCs. Delaying administration of MSCs permits initial inflammatory response to resolve, allowing adequate nutrient/oxygen supply which favors the survival of MSCs, thus promoting healing. The concept of delayed MSC therapy has also been found to be effective in rats with diabetic renal injury and goats with defective maxilla [30, 31]. These novel findings suggest that delayed application is a new approach for transplanting MSC-EVs. MSC-EVs have been found to have similar reparative properties (immune suppression, proangiogenesis, and proosteogenic) as MSCs, and these properties of MSC-EVs are partly mediated by the cross-talk between MSC-EVs and their target cells [32, 33]. After fat transplantation, several donor cells might die within few weeks. Cells in the recipient migrate and participate in the regeneration of graft tissue [34]. This implies that EVs may be internalized by more HUVECs if they are administered late. We hypothesized that delayed supplementation will improve the regeneration potential of these vesicles.

Fat grafting is widely applied in face rejuvenation, treatment of scars, breast reconstruction, hand contractures, and hemifacial atrophy among other diseases [35]. Previous studies have demonstrated that transplantation of premixed ADSC-EVs/fat promotes angiogenesis of fat transplants by enhancing proliferation and migration of endothelial cells [36]. Increased neovascularization may also improve maintenance of the graft volume. Moreover, in clinical treatment, fat is often grafted on superficial areas (dermal and subdermal layers), which are suitable for delayed injection of MSC-EVs after grafting.

**Figure 1:**
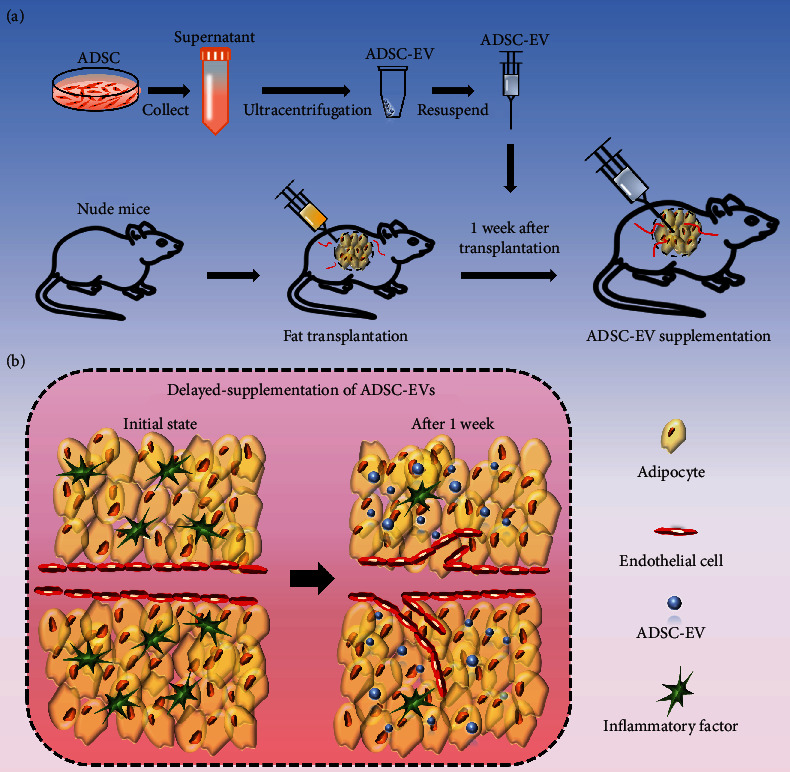
Schematic representation of experimental design and its potential mechanism. (a) ADSCs were cultured and the supernatant was collected. ADSC-EV was obtained by ultracentrifugation technique. Subsequently, we subcutaneously injected human fat tissue into nude mice followed by ADSC-EV injection at 1 week after fat grafting. (b) The scheme shows that, at 1-week after grafting, endothelial cells derived from the recipient are recruited to form the new vessels in transplants and the inflammatory reaction subsides, making the environment more conducive for ADSC-EV to display its regenerative effects.

Accordingly, we devised a novel strategy of delayed injection of MSC-EV for the regeneration of transplanted tissues. As shown in Figure 1(a), ADSC-EVs were isolated from the supernatant of cultured ADSCs using ultracentrifugation techniques. Subsequently, human fat tissue was subcutaneously injected into nude mice. One week later, ADSC-EVs were injected into the fat graft. Histological and immunohistochemical analyses were performed 3 months posttransplantation to assess vascularization and inflammation reactions in the fat grafts. Potential mechanisms for the proangiogenesis effects of ADSC-EVs were investigated with RNA deep sequencing.

## 2. Materials and Methods

### 2.1. Extraction of Human Fat Tissue

Fat tissues were obtained from female patients (20-40 years old) who underwent abdominal liposuction at the Department of Plastic Surgery, Wuhan Union Hospital. A written informed consent was obtained from each patient before sample collection. The study protocol was approved by the Ethics Committee of Tongji Medical College (approval number: 2019-S069), and this experiment was conducted in accordance with the Declaration of Helsinki.

### 2.2. Isolation and Characterization of ADSCs

#### 2.2.1. Isolation

ADSCs were isolated according to the procedure we introduced before with some modifications. Briefly, lipoaspirates were washed in phosphate-buffered saline (PBS) for three times and then treated with 0.1% (wt/vol) collagenase at 37°C for 1.5 hours. The suspension was centrifuged (280 g, 5 min), and the pellet was resuspended in culture medium, containing low-glucose Dulbecco's modified Eagle medium supplemented (L-DMEM), 10% fetal bovine serum (FBS) which was from Hyclone (GE Healthcare Life Science, Logan, UT, USA), and 1% penicillin-streptomycin. After that, the fraction was seeded in 10 cm petri dishes and incubated at 37°C/5% CO_2_.

#### 2.2.2. Multilineage Differentiation of ADSCs

The differentiation capacity of ADSCs was identified as previously described [31]. All chemicals were purchased from Sigma-Aldrich (St. Louis, MO, USA) unless specifically stated. To investigate the osteogenic differentiation ability of ADSCs, cells were incubated in osteogenic medium (L-DMEM, 10% FBS, 1% penicillin-streptomycin, 10 mM *β*-glycerophosphate, 50 *μ*M ascorbic acid, and 0.1 *μ*M dexamethasone) for 2 weeks, and then, Alizarin Red staining was performed to identify extracellular matrix calcification. To inspect the adipogenic differentiation potential of ADSCs, adipogenic medium (made up of high-glucose Dulbecco's modified Eagle medium (HDMEM), 10% FBS, 1% penicillin-streptomycin, 0.5 mM 3-isobutyl-1-methylxanthine, 10 *μ*M insulin, 200 *μ*M indomethacin, and 1 *μ*M dexamethasone) was used. Cells were induced for 3 weeks and then were assessed by oil red O staining. For chondrogenic differentiation, chondrogenic medium was used (L-DMEM, 10% FBS, 1% penicillin-streptomycin, 1 mM sodium pyruvate, 1% insulin-transferrin sodium-selenite, 0.17 mM ascorbic acid, 0.35 mM L-proline, 1.25 mg/mL bovine serum albumin, 5.33 *μ*g/mL linoleic acid, 0.1 *μ*M dexamethasone, and 0.01 *μ*g/mL transforming growth factor-*β* (Cell Science, Canton, MA, USA)). After 4-week induction, Toluidine Blue staining was performed.

#### 2.2.3. Immunophenotyping

ADSCs were fixed in 4% paraformaldehyde for 30 min and then washed with PBS. The primary antibodies applied were mesenchymal stromal cell markers (CD44, CD45, CD90, CD29, and CD105) of Antibody Panel (Cat. ab93758; Abcam, Cambridge, UK) which includes rabbit anti-human CD29 (1 : 100), rabbit anti-human CD45 polyclonal monoclonal antibodies (1 : 100), mouse anti-human CD44, CD90, and CD105 monoclonal antibodies (1 : 100). Secondary antibodies used were goat anti-rabbit IgG conjugated with fluorescein isothiocyanate (FITC) and rabbit anti-mouse IgG conjugated with FITC, respectively. 4,6-Diamidino-2-phenylindole (DAPI) was used to identify nuclear.

### 2.3. Isolation and Characterization of ADSC-EVs

#### 2.3.1. Isolation

ADSC-EVs were isolated as previously described but with minor modifications [32]. Briefly, when ADSCs reached 70–80% confluence after 4 to 6 passages, the culture medium was replaced by serum-free culture medium. Culture supernatants collected after 24 hours were first centrifuged at 700 g for 15 min, and the precipitate was discarded. Second centrifugation was performed at 2000 g for 20 min, and precipitate was discarded as well. The supernatant was further centrifuged at 16,000 g, 4°C for 1 h. The pellet containing ADSC-EVs was resuspended in PBS and then centrifuged at 16,000 g, 4°C for 1 h. The pellet was resuspended in 1 mL PBS for further analyses. The protein content of ADSC-EVs was measured using bicinchoninic acid (BCA) assay (Thermo Scientific, Waltham, MA, USA).

#### 2.3.2. Transmission Electron Microscopy (TEM)

10 *μ*L ADSC-EVs (the concentration of ADSC-EVs used in this experiment was 20 *μ*g/mL) were first added to a formvar-carbon-coated grid. After ADSC-EVs had been allowed to be adhered to the grid for 30 min, the grid was rinsed with droplets of deionized water and fixed for 5 min with 1% glutaraldehyde. The sample was then stained with 2% uranyl acetate. After removing remnant liquid using a blotting paper, the grid was dried at room temperature. Photographs were taken using a HT7700 microscope (Hitachi, Tokyo, Japan) at an acceleration voltage of 80 kV.

#### 2.3.3. Confocal Microscopic Analysis

The ADSC-EV resuspension was centrifuged for 1 h at 16000 g, under 4°C, and the resultant pellet was resuspended in 1 mL of Diluent C (for General Membrane Labeling). ADSC-EVs were then labeled using 4 *μ*L (1 mM) of PKH26 red fluorescent cell linker for 30 min at 37°C (Sigma-Aldrich, St Louis, MO, USA) following the manufacturer's instructions. After incubation, the sample was centrifuged at 16000 g for 1 h at 4°C. The pelleted PKH26 red fluorescent-labeled ADSC-EVs were observed under an A1Si confocal microscope (Nikon, Tokyo, Japan).

#### 2.3.4. Nanoparticle Tracking Analysis

A nanosight NS300 with a 405 nm laser (Malvern, Malvern, UK) was used to identify the size distribution and concentration of ADSC-EVs. The concentration of ADSC-EVs used in this experiment was 5 *μ*g/mL (2.78 × 10^9^ particles/mL). The analysis was performed at room temperature.

#### 2.3.5. Western Blotting

Radioimmunoprecipitation assay buffer cooled to 4°C was added to ADSC-EV pellet. The pellet was lysed on ice for 30 min. BCA protein assay was performed to quantify the concentration of proteins in ADSC-EVs following the manufacturer's protocol. The proteins were resolved by acrylamide gel electrophoresis. After electrophoresis, the protein gels were rinsed with water immediately. Then, protein was transferred to polyvinylidene difluoride membranes. The membranes were blocked with 5% nonfat dry milk in PBS and 0.05% Tween 20 and incubated overnight with the following primary antibodies: rabbit anti-TSG101 antibody (Cat. GB11618; Servicebio, Wuhan, China) and rabbit anti-VEGF antibody (Cat. 19003; Chicago, USA). The membranes were incubated with horseradish peroxidase-conjugated goat anti-rat IgG (1 : 2000; Abcam) for 1 h. Amersham Hyperfilm™ ECL was applied to visualize the blots.

### 2.4. Cellular Uptake of ADSC-EVs

ADSC-EVs were labeled with PKH26 red fluorescent cell linker as described in the preceding section. HUVECs obtained from American Type Culture Collection (Rockville, MD, USA) were plated on coverslips. HUVECs were incubated with 20 *μ*g/mL (11.12 × 10^9^ particles/mL) of ADSC-EVs for 4 hours. The samples were washed three times with PBS and fixed for 30 min in 4% paraformaldehyde. HUVECs were stained with 4′,6-diamidino-2-phenylindole (DAPI). Finally, photography was performed using structured illumination microscopy (Nikon, Tokyo, Japan).

### 2.5. Scratch Assay

Briefly, 1 × 10^4^ HUVECs/well were seeded in a 48-well plate and incubated in L-DMEM supplemented with 10% FBS and 1% penicillin-streptomycin. When HUVECs grew to 90-100% confluence, a cross-shaped scratch was made in the field of cells using a 200 *μ*L pipette tip. HUVECs were gently washed with PBS to remove cellular debris. The cells were cultured either in serum-free culture medium containing varied concentration of ADSC-EVs (the final concentration of ADSC-EVs was 10, 20, or 40 *μ*g/mL, respectively) or PBS (control). Finally, photographs were taken at 0, 6, 12, and 18 h using a phase-contrast microscope (Nikon, Tokyo, Japan). Change in the width of the wounds was measured using the Image-Pro Plus 6. The experiments were repeated 3 times under the same conditions.

### 2.6. Tube Formation Assay

Growth factor-reduced Matrigel (BD Bioscience, San Jose, CA, USA) was thawed at 4°C. A 96-well culture plate was precoated with 50 *μ*L Matrigel/well. The plate was then incubated at 37°C for 30 min to solidify the Matrigel. HUVECs were divided into four groups and pretreated with serum-free medium supplemented with either ADSC-EVs (the final concentration of ADSC-EVs was 10, 20, or 40 *μ*g/mL, respectively) or PBS (control) for 24 hours at 37°C. The pretreated HUVECs were seeded in a 96-well culture plate (1 × 10^4^ of HUVECs per well; three wells/group), followed by a 6-hour incubation at 37°C under 5% CO_2_. Finally, photography was performed using a phase-contrast microscope. The length of the tube (fold change) and the number of vessel-like structures were assessed with an Image-Pro Plus 6. The experiments were repeated 3 times under the same conditions.

### 2.7. Fat Grafting on the Nude Mouse Model

The experiment was approved by the Ethical Review Committee of Tongji Medical College, Huazhong University of Science and Technology. The protocol was approved by ethical committee (approval number: 2019-S069). Lipoaspirate was obtained from a female patient (25 years old) who underwent abdominal liposuction at Wuhan Union hospital. Twenty-four, 6-week-old BALB/c nude mice weighing 16-20 g were obtained from Beijing Vital River Laboratory (Animal Technology Co. Ltd, Beijing, China). The mice were randomly divided into three groups: Blank, EV-immediate, and EV-delayed groups (*n* = 8). The mice were anesthetized with 3% inhaled isoflurane. A mini-incision was first created in the middle of the backs of the mice, and two tunnels were created by passing an 18-gauge needle beneath the subcutaneous layer. Two sites per mouse were subcutaneously transplanted with 0.35 mL of human fat using an 18-gauge needle. During the process of fat transplantation, a subcutaneous pocket was made by the needle to restrain the leakage of fat tissue. Every fat graft in control and EV-delayed group received a 100 *μ*L PBS injection, whereas each site in the immediate group received 7 *μ*g (about 3.9 × 10^9^ particles) of ADSC-EVs suspended in 100 *μ*L PBS. The incisions were sutured with 6/0 nylon. One week after fat grafting, each graft in the control and EV-immediate groups was injected with 100 *μ*L PBS, and each point in the EV-delayed group was injected with 7 *μ*g of ADSC-EVs suspended in 100 *μ*L PBS. Four mice in each group were sacrificed and the fat grafts isolated for subsequent analyses at 1 and 3months postfat transplantation.

### 2.8. Graft Weight and Volume Measurements

Each graft was photographed and weighed. The volume of each fat graft was determined with the liquid overflow method. Briefly, each sample was immersed in a syringe full of PBS. The displaced volume was equivalent to the volume of the tissue.

### 2.9. Hematoxylin and Eosin Analyses

The transplants were placed in 4% paraformaldehyde for 24 h and then embedded in paraffin. Tissue sections from the middle of each graft were stained with hematoxylin and eosin. Images of the sections were captured using a light microscope (Nikon, Tokyo, Japan). The diameter of adipocytes was analyzed with ImageJ. The tissues were independently assessed twice in a double-blinded manner.

### 2.10. Immunohistological Analyses

Immunofluorescent staining was performed using Perilipin-1 (D1D8) XP® Rabbit mAb (Alexa Fluor® 488 Conjugate) (Cat. 29138s; Cell Signaling Technology, Danvers, USA) according to the manufacturer's protocol.

For immunohistochemical staining, paraffin-embedded fat graft sections (8 mm thick) were relatively incubated with rabbit polyclonal to CD206 (Cat. ab64693), rabbit anti-human Ki67 (Cat. ab15580), and rabbit anti-human CD31 (Cat. ab28364) antibodies overnight at 4°C. After washing with PBS containing 0.1% Tween 20 (PBST), the sections were incubated with a horseradish peroxidase-conjugated goat anti-rabbit secondary antibody (Proteintech Group, Wuhan, China) for 30 min at room temperature. The sections were colorized with 3,3′-diaminobenzidine tetrahydrochloride for 3 min at room temperature. Five randomly selected fields were captured under a light microscope at ×20 magnification (Nikon, Tokyo, Japan). The number of CD206, Ki67-positive cells, and CD31-positive vessels was calculated by two independent reviewers.

### 2.11. RNA Extraction, Library Preparation, and Deep Sequencing

Total RNA was extracted from extracellular vesicles using Trizol (Invitrogen, Carlsbad, CA). Contaminating DNA was digested using DNaseI. The quality of RNA was evaluated using a 260/A280 Nanodrop™ OneC spectrophotometer (Thermo Scientific, Waltham, MA, USA). The integrity of the RNA was assessed using 1.5% agarose gel electrophoresis. To prepare RNA library, 3 *μ*L of total RNA was subjected to Next Small RNA Library Prep Set for Illumina (NEB) in line with the manufacturer's instructions. Briefly, RNA was used as the input for RNA adapter ligation (using 3′ and 5′ RNA adapters) and then reverse-transcribed to create cDNA templates for PCR amplification. The PCR products were pooled (equal volumes) and used for size selection on a Pippin Prep (Sage Science, USA) to recover the fractions containing mature miRNAs. The resulting small RNA libraries were concentrated by ethanol precipitation and quantified with Qubit 3.0 with a Qubit™ RNA Broad Range Assay (Thermo Scientific, Waltham, MA, USA) prior to sequencing on a NextSeq 500 sequencer (Illumina, USA).

### 2.12. miRNA-Seq Data Analysis

Raw sequencing data were first filtered using fastx_toolkit (version 0.0.13.2) to discard low-quality reads. Adaptor sequences were trimmed using Cutadapt (version 1.15). Clean reads from each sample were then mapped onto the reference genome of *Homo sapiens* (*Homo_sapiens*. GRCh38; ftp://ftp.ensembl.org/pub/release-87/fasta/homo_sapiens/dna/) using bowtie (version: 1.1.2) under default parameters. Mirdeep2 (version 2.0.0.8) package was used to map the reads to known primary miRNAs in the miRBase database, as well as predicting novel miRNAs. Differentially expressed miRNAs between the groups were identified using the edgeR package (version 3.12.1) based on *P* < 0.05 and |Log_2_ fold change| > 1 as the cut-offs of statistical significance. Target mRNAs for the differentially expressed miRNAs were predicted using Miranda v 3.3a. Gene ontology (GO) analysis for the target mRNA was performed using KOBAS software (version 2.1.1) with a corrected *P*˂0.05 considered statistically significant.

### 2.13. Statistical Analysis

Data analysis was performed with Graphpad Prism 5 (GraphPad Software Inc.). The data were presented as the mean ± standard deviation (SD). Differences between the means of groups were compared using one-way analysis of variance (ANOVA) and Tukey's test. Statistical significance was set at *P* < 0.05.

## 3. Results

### 3.1. ADSC Characterization

Oil red O staining revealed neutral lipid droplets in the ADSC cytoplasm after adipogenesis induction (Figure S1A). Alizarin Red staining showed extracellular calcium elevation after osteogenesis induction (Figure S1A). Toluidine blue staining revealed that chondrogenic induction initiated chondrogenic differentiation (Figure S1A), while immunofluorescence staining indicated that the ADSCs were positive for CD90 and CD105 (mesenchymal stem cell markers) and CD29 and CD44 (cell adhesion molecules) but not CD45 (hematopoietic marker) (Figure S1B).

### 3.2. ADSC-EV Characterization and Internalization

**Figure 2:**
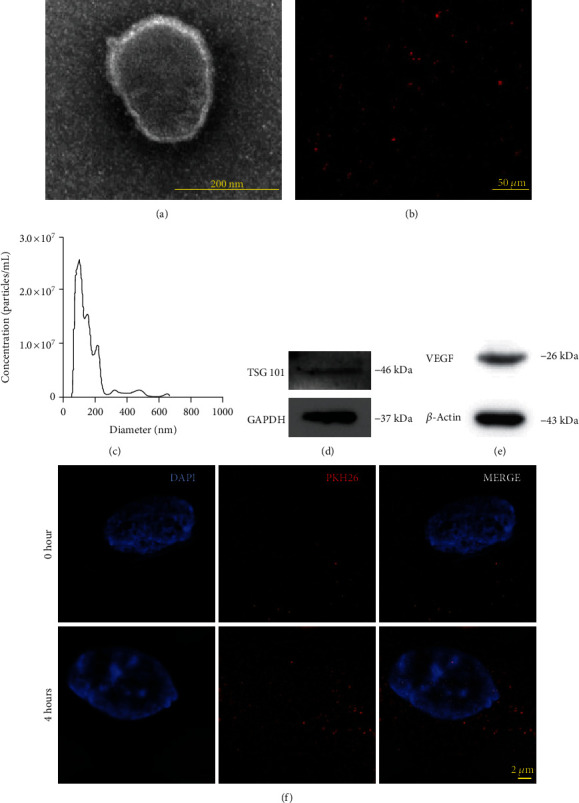
Identification and internalization of ADSC-EVs. (a) Transmission electron microscopy image of ADSC-EV after negative staining shows EVs are on average about 200 nm. Scar bar = 200 nm. (b) Picture of ADSC-EVs labeled with the red fluorescent dye PKH26. Scar bar = 50 *μ*m. (c) Nanosight profile of ADSC-EVs. Their sizes and concentrations are shown. Diameter distribution peaks at around 166 nm. (d) Western blot results showing positive expression of TSG101 (EV surface markers) and (e) VEGF in ADSC-EVs. (f) PKH26-labelled ADSC-EVs (red fluorescence) were added to HUVECs (nuclei were stained by DAPI); representative structured illumination microscopy images are shown. Scar bar = 2 *μ*m.

About 100 *μ*g ADSC-EVs were collected from a 320 mL supernatant, requiring about 50 million ADSCs. TEM (Figure 2(a)) and A1Si confocal microscope (Figure 2(b)) indicated that ADSC-EVs were spherical and surrounded by a lipid bilayer. Nanoparticle tracking analysis showed that ADSC-EVs were highly heterogeneous (about 100 to 600 nm) with a peak of 166 nm (Figure 2(c)). ADSC-EVs expressed TSG101 (Figure 2(d)) and VEGF (Figure 2(e)). Structured illumination microscopy showed that red fluorescence surrounded the ADSC-EV-incubated HUVECs, indicating that HUVECs internalized ADSC-EVs (Figure 2(f)).

### 3.3. ADSC-EVs Promote HUVEC Migration and Tube Formation In Vitro

**Figure 3:**
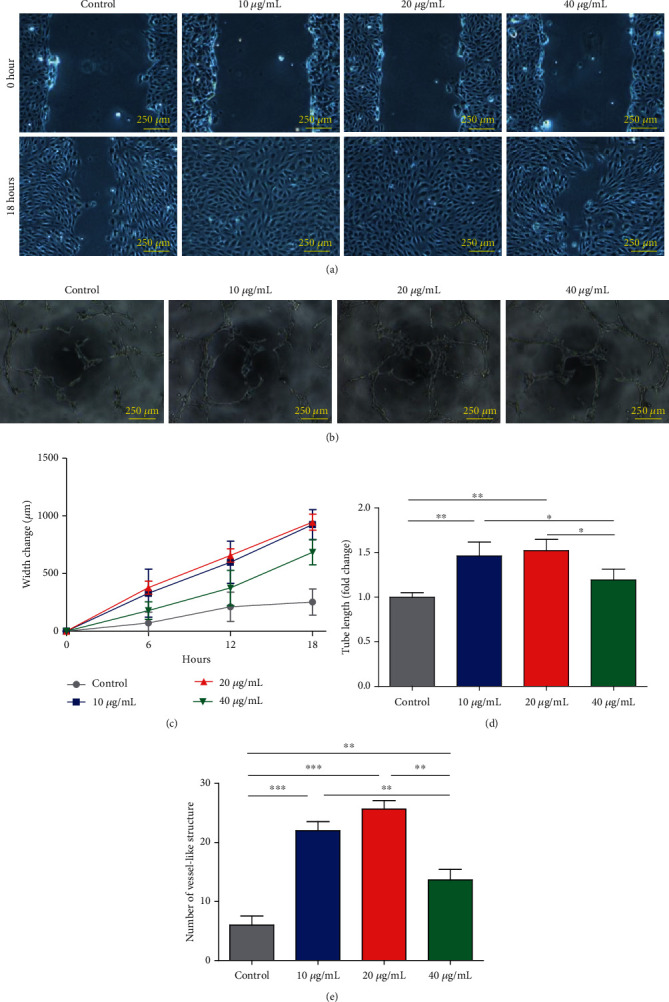
ADSC-EVs promoted the migration and tube formation activities of HUVECs. Scratch assay (a) and tube formation assay (b) of HUVECs after treatments with PBS, 10 *μ*g/mL, 20 *μ*g/mL, and 40 *μ*g/mL ADSC-EVs. Representative images of each group are shown. Scar bar = 250 *μ*m. The width change (c), tube length (d), and number of vessel-like structure (e) of each group are shown. *n* = 6, 3, and 3 for width change, tube length, and vessel-like structure, respectively. ^∗^*P* < 0.05,  ^∗∗^*P* < 0.01, and^∗∗∗^*P* < 0.001.

Scratch (Figure 3(a)) and tube formation assays (Figure 3(b)) were performed to examine ADSC-EV angiogenesis properties *in vitro*. The wound width significantly changed after 10 and 20 *μ*g/mL ADSC-EV treatments (control: 253.12 ± 113.67 *μ*m; 10 *μ*g/mL: 924.59 ± 129.84 *μ*m; 20 *μ*g/mL: 945.02 ± 69.41 *μ*m; 40 *μ*g/mL: 684.41 ± 109.29 *μ*m; at 18 hours; Figure 3(c)). Similarly, ADSC-EV supplementation increased the vessel-like structure formation. The folding tube length increased (control: 1.00 ± 0.05; 10 *μ*g/mL: 1.46 ± 0.16; 20 *μ*g/mL: 1.52 ± 0.12; 40 *μ*g/mL: 1.19 ± 0.12; Figure 3(d)) and number of vessel-like structures also increased (control: 6.00 ± 2.65; 10 *μ*g/mL: 22.00 ± 2.65; 20 *μ*g/mL: 25.67 ± 2.52; 40 *μ*g/mL: 13.67 ± 3.02; Figure 3(e)) in both 10 and 20 *μ*g/mL groups, indicating the proangiogenic potential of ADSC-EVs.

### 3.4. Quantitative Analysis of Fat Grafts

**Figure 4:**
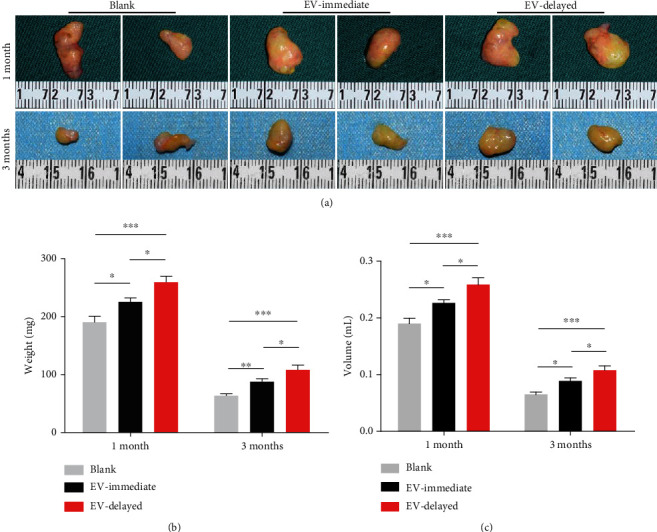
*In vivo* fat grafting with delayed injection of ADSC-EVs. (a) Photographs of fat grafts taken from different groups of nude mice after 1 and 3 months. Quantitative analysis of (b) weight and (c) volume from mice after different treatments. *n* = 8 for both. ^∗^*P* < 0.05 and^∗∗∗^*P* < 0.001.

Fat graft images in each group (at months 1 and 3 after transplantation) are shown in Figure 4(a). Fat liquefaction occurred in the Blank group grafts. At month 3, the average fat graft weights in the Blank, EV-immediate, and EV-delayed groups were 63.53 ± 9.38 mg, 87.80 ± 13.39 mg, and 108.05 ± 21.97 mg, respectively, while the volumes were 0.06 ± 0.01 mL, 0.08 ± 0.01 mL, and 0.11 ± 0.02 mL, respectively. Therefore, the delayed EV introduction has a significant effect (Figures 4(b) and 4(c)).

### 3.5. Histochemical Analyses

**Figure 5:**
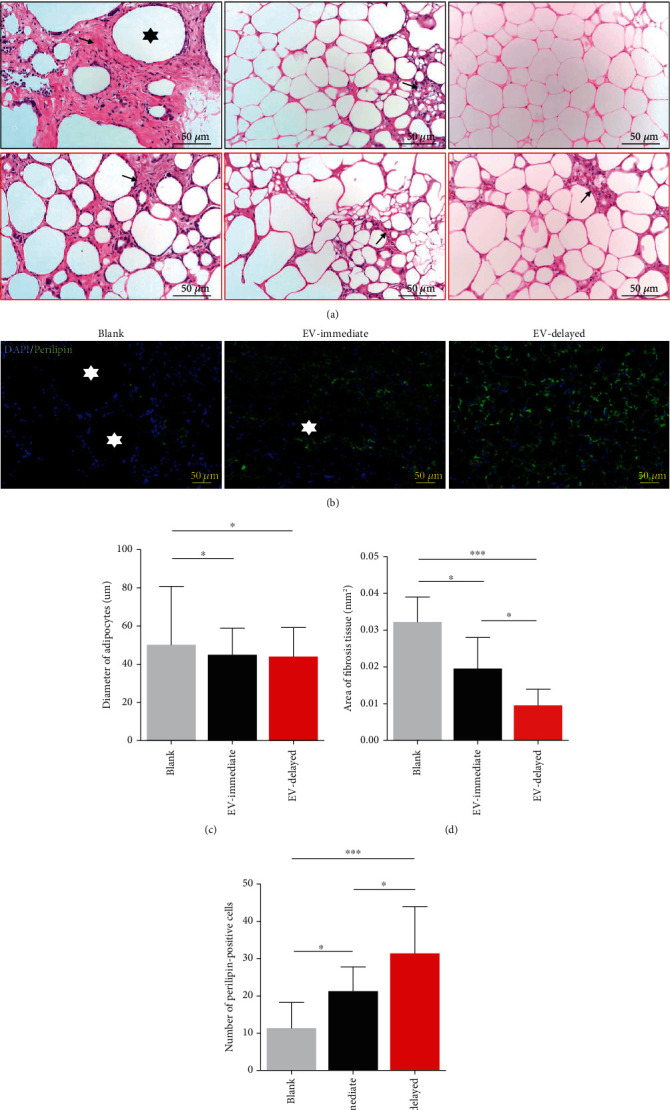
Histological evaluation and immunofluorescence staining of fat grafts after 3 months of transplantation. (a) H&E-stained pictures (low magnification and high magnification) and (b) immunofluorescence stained images (perilipin: green fluorescence) of grafts slices taken from three groups at month 3. Black arrows represent fibrosis and black hexagrams indicate vacuoles. White hexagrams show perilipin-negative crown-like structures. Quantitative analysis of (c) diameter of adipocytes, (d) area of fibrosis tissue, and (e) number of per perilipin-positive cells. ^∗^*P* < 0.05 and^∗∗∗^*P* < 0.001.

There were lower graft cysts levels in EV-immediate and EV-delayed groups after three months of transplantation (Figure 5(a), black hexagram). Additionally, the fat cells were more homogeneous in the EV-immediate and EV-delayed transplant groups than in the Blank group (adipocyte diameters in Blank, EV-immediate, and EV-delayed groups were 50.17 ± 30.50 *μ*m, 44.91 ± 14.01 *μ*m, and 43.99 ± 15.20 *μ*m, respectively) (Figure 5(c)). Fibrosis was least in the EV-delayed group, compared with the Blank and EV-immediate groups (Blank group: 0.032 ± 0.006 mm^2^, EV-immediate group: 0.020 ± 0.008 mm^2^, and EV-delayed group: 0.010 ± 0.004 mm^2^) (Figure 5(d)). The EV-delayed group had the fewest graft perilipin-negative crown-like structures (Figure 5(b), white pentagram). Quantitative analysis indicated that the number of perilipin-positive cells was highest in the EV-delayed group (Blank group: 11.38 ± 6.46 mm^2^, EV-immediate group: 21.25 ± 6.12 mm^2^, and EV-delayed group: 31.38 ± 11.74 mm^2^) (Figure 5(e)).

**Figure 6:**
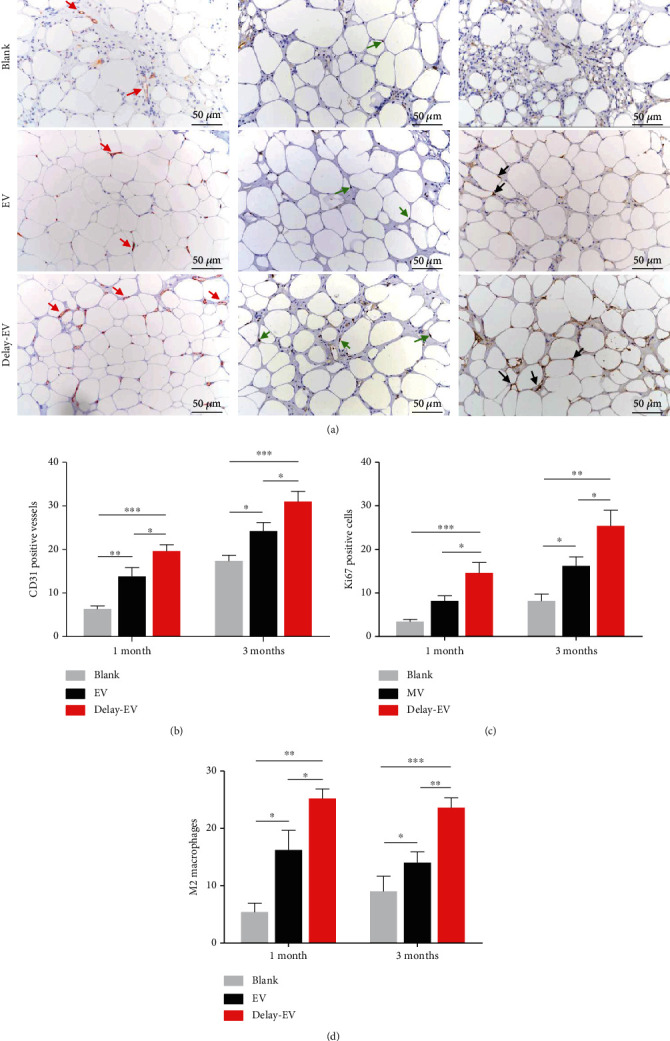
Immunohistochemical staining for fat transplants after 3 months. (a) Immunohistochemical staining images (CD31, Ki67, and CD206) of grafts slices taken from three groups at month 3. Scar bar = 50 *μ*m. Quantitative analysis of (b) CD31-positive vessels, (c) Ki67-positive cells, and (d) M2 macrophages of each group (all *n* = 5). ^∗^*P* < 0.05,  ^∗∗^*P* < 0.01, and^∗∗∗^*P* < 0.001.

The representative image of each group on CD31, Ki67, and CD206 analyses is shown in Figure 6(a). Herein, the EV-delayed group had the highest number of CD31-positive vessels (Blank group: 17.40 ± 2.58, EV-immediate group: 24.20 ± 3.97, and EV-delayed group: 31.00 ± 4.60). Similarly, Ki67 positive cells (Blank group: 8.20 ± 3.12, EV-immediate group: 16.20 ± 4.17, and EV-delayed group: 25.40 ± 7.14) and CD206-positive M2 macrophages cells (Blank group: 9.00 ± 5.33, EV-immediate group: 14.00 ± 3.85, and EV-delayed group: 23.60 ± 3.44) were highest in the EV-delayed group (Figures 6(b)–6(d)).

### 3.6. ADSC-EVs Transfer Functional miRNAs to Target Cells

**Figure 7:**
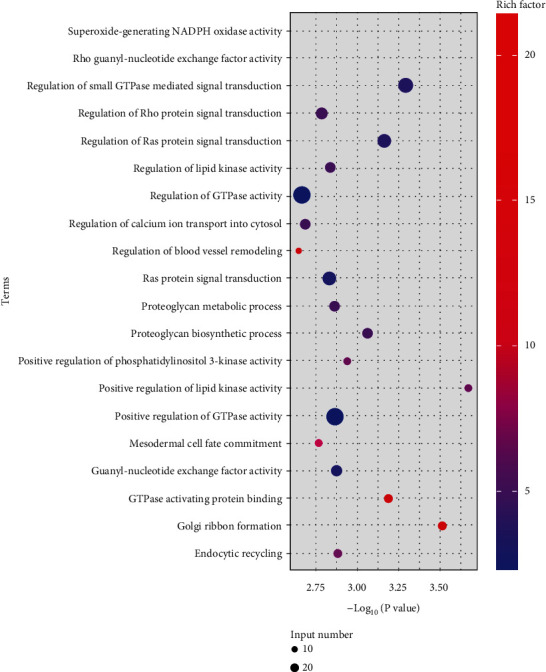
Potential proangiogenic ability of ADSC-EVs. Top 20 (according to *P* value) GO terms for miRNA target genes. The enrichment factor represents the ratio between the total number of genes in the input list and the number of annotated genes in the GO terms. The larger the enrichment factor, the higher the level of enrichment.

Previous studies have shown that EVs transport miRNAs to target cells. Therefore, the ADSC-EV-miRNAs were sequenced; then (Gene Ontology) GO enrichment analyses were conducted. The top 20 enriched GO terms in ADSC-EVs are shown in Figure 7. The miRNAs were associated with several biological processes. The top 20 enriched GO terms included the blood vessel remodeling regulation. miR-21, miR-92, miR-320, miR-27, miR-221, and miR-126, involved in angiogenesis, were among the top 50 most enriched miRNAs in ADSC-EVs (Supplementary Table 1) [37].

## 4. Discussion

The delayed-supplementation strategy was used following the dynamic remodeling of adipose tissue after fat grafting to improve the proregenerative efficiency of ADSC-EVs. Three peripheral zones surrounded the graft after transplantation: the surviving zone (adipocytes survived), the regenerating zone (dead adipocytes were replaced with new ones), and the necrotic zone (both adipocytes and ADSCs died) [34]. It was reported that most adipocytes (except for those located superficially) die as early as day 1 after fat grafting. The number of proliferating cells in fat grafts increased from day 3, and an increase in viable adipocyte area was observed from day 7, indicating repair/regeneration of the dead tissue [34]. Delayed MSC-EV injection into the regeneration zone replenished the angiogenic growth factors (miRNA encapsulated in ADSC-EVs) in fat regeneration. It prevented immune rejection at early transplantation, enhancing angiogenesis at the peak and maintaining it for longer. Therefore, ADSC-EV injection on day 7 after grafting (coordinating with the self-repair of the fat grafts) can promote ADSC-EV effectiveness, thus enhancing interior fat tissue regeneration and increasing the final volume retention rate.

MSC-EVs have proangiogenic functions. In this study, proangiogenic properties were confirmed in ADSC-EVs. Mounting evidence has shown that miRNAs translocated by EV have essential physiological roles [38, 39], such as regulating gene expression and performance of target cells [3]. For instance, human monocyte/macrophage cell line THP-1 EVs transfer miR-150 to human microvascular endothelial cells (HMEC). Exogenous miR-150 regulates c-Myb expression, improving HMEC migration [40]. In this study, microRNAs (miR-21, miR-92, miR-320, miR-27, miR-221, and miR-126) with angiogenic functions were detected among the top 50 most expressed microRNAs in ADSC-EVs (Supplementary Table 1) [37]. Therefore, ADSC-EVs transfer proteins and miRNAs to endothelial cells, modifying the cell physiology, thus improving the neoangiogenesis in fat grafts. However, further studies are needed to describe the precise molecular mechanisms involved.

Perilipin on the intracellular lipid droplet surface regulates triglyceride accumulation and hydrolysis [41]. One study reported that perilipin gene expression increased early and remained stable during pre-adipocyte differentiation [42]. Therefore, high perilipin-positive cell levels in the EV-delayed group indicated functional adipocytes. Immunohistochemical analyses (Figure 6) showed that CD31-positive cells were highest in the EV-delayed group (Figure 6(b)). Previous studies have demonstrated that ischemia and hypoxia inhibit cell survival in fat grafts, destroying the nucleus and cellular membranes, causing the development of fatty cysts, and killing fat tissue [43]. Therefore, improved neovascularization is positively associated with fat retention. However, it is negatively associated with fibrosis tissue formation.

Delayed ADSC-EV treatment might enhance tissue regeneration by inducing the proliferation of recruited cells (stem cells, endothelial cells) or survived cells. Meanwhile, M2 macrophages secrete proangiogenic and anti-inflammatory factors, such as bFGF, VEGF, and IL-10, which provide a permissive microenvironment for cell survival [44, 45]. M2 macrophages also secrete soluble factors that drive proliferation and differentiation of pluripotent ADSCs into mature lipid-laden adipocytes in fat grafts [46]. Therefore, both the number of perilipin-positive cells and the graft retention volume of EV-delayed group increased.

The number of CD31-positive vessels was highest in the EV-delayed group, indicating that ADSC-EV supplementation is efficient 1-week postgraft transplantation. A study showed that most donor cells die within 2 weeks after fat grafting. Cells moved and participated in graft tissue regeneration after one to eight weeks of transplantation [47]. The endothelial cells were recruited to form new vessels in the transplants after one grafting week (Figure 1(b)). Therefore, ADSC-EVs have more target cells. Besides, the inflammatory reaction decreased, providing a more permissive graft environment for recruited endothelial cells.

Several studies have indicated that MSC supplementation improves graft fat retention. However, the retention after preenrichment with ADSCs and ADSC-EVs was not compared. They had different concentrations, making it difficult to determine their equivalent dose in the fat transplantation model [48].

It is worth mentioning that HUVECs added with 40 *μ*g/mL of EVs had no significant differences in cell migration compared with the control group (Figure 3). It was reported previously that EVs could inhibit cell migration and tube formation through a CD36-mediated mechanistic pathway [18]. Meanwhile, EVs contain substance which may positively regulate cell migration [19]. Therefore, it is possible that EVs display positively or negatively effects on cell migration at different concentrations.

## 5. Conclusion

In conclusion, delayed ADSC-EV treatment (a week after fat grafting) yields better results than immediate injection. This study can improve the understanding of the potential clinical value and use of MSC-EVs. Besides, the delayed injection approach can be effective in other tissue repairs.

## Data Availability

The data that support the findings of this study are available from the corresponding authors upon request.

## Abbreviations

MSC: Mesenchymal stromal cells
EV: Extracellular vesicles
MSC-EV: Extracellular vesicle from mesenchymal stromal cells
ADSC-EV: Extracellular vesicles from adipose-derived stem cells
BCA: Bicinchoninic acid
DAPI: 4,6-Diamidino-2-phenylindole
PBS: Phosphate-buffered saline
HUVEC: Human umbilical vein endothelial cell
L-DMEM: Low-glucose Dulbecco's modified Eagle medium
HMEC: Human microvascular endothelial cell
TEM: Transmission electron microscopy
TSG 101: Tumor susceptibility 101
VEGF: Vascular endothelial growth factor.

## Supplementary Materials

Supplementary 1 Supplemental table 1: miRNAs involved in angiogenesis among the top 50 most enriched miRNAs in ADSC-EVs.

Supplementary 2 Figure S1: characteristics of ADSCs.
