# Supplementary material for: Delayed Supplementation Strategy of Extracellular Vesicles from Adipose-Derived Mesenchymal Stromal Cells with Improved Proregenerative Efficiency in a Fat Transplantation Model
Source: Stem Cells Int. 2022 Sep 7;2022:2799844. doi: 10.1155/2022/2799844 (PMC9476248; doi:10.1155/2022/2799844)
Supplement: Supplementary 1 — Supplemental table 1: miRNAs involved in angiogenesis among the top 50 most enriched miRNAs in ADSC-EVs. [file 2799844.f1.docx]

Supplemental table 1. miRNAs involved in angiogenesis among the Top 50 most enriched miRNAs in ADSC-EVs

| miRNAs | Angiogenic Function | Relevant targets |
| --- | --- | --- |
| miR-21 | Required for viral protein induced endothelial cell migration | ? |
| miR-92 | overexpression in tumor cells promotes tumor angiogenesis | TSP-1 CTGF |
| miR-320 | Inhibition of miR-320 improves angiogenesis in diabetic endothelial cell | IGF-1 |
| miR-27 | Required for angiogenesis in vitro | ? |
| miR-221 | Impairs SCF induced angiogenesis | c-Kit |
| miR-126 | Required for vascular integrity and angiogenesis in vivo | Spred-1, PIK3R2 |
