# Supplementary material for: Delayed Supplementation Strategy of Extracellular Vesicles from Adipose-Derived Mesenchymal Stromal Cells with Improved Proregenerative Efficiency in a Fat Transplantation Model
Source: Stem Cells Int. 2022 Sep 7;2022:2799844. doi: 10.1155/2022/2799844 (PMC9476248; doi:10.1155/2022/2799844)
Supplement: Supplementary 2 — Figure S1: characteristics of ADSCs. [file 2799844.f2.docx]

**Supplemental Figures**


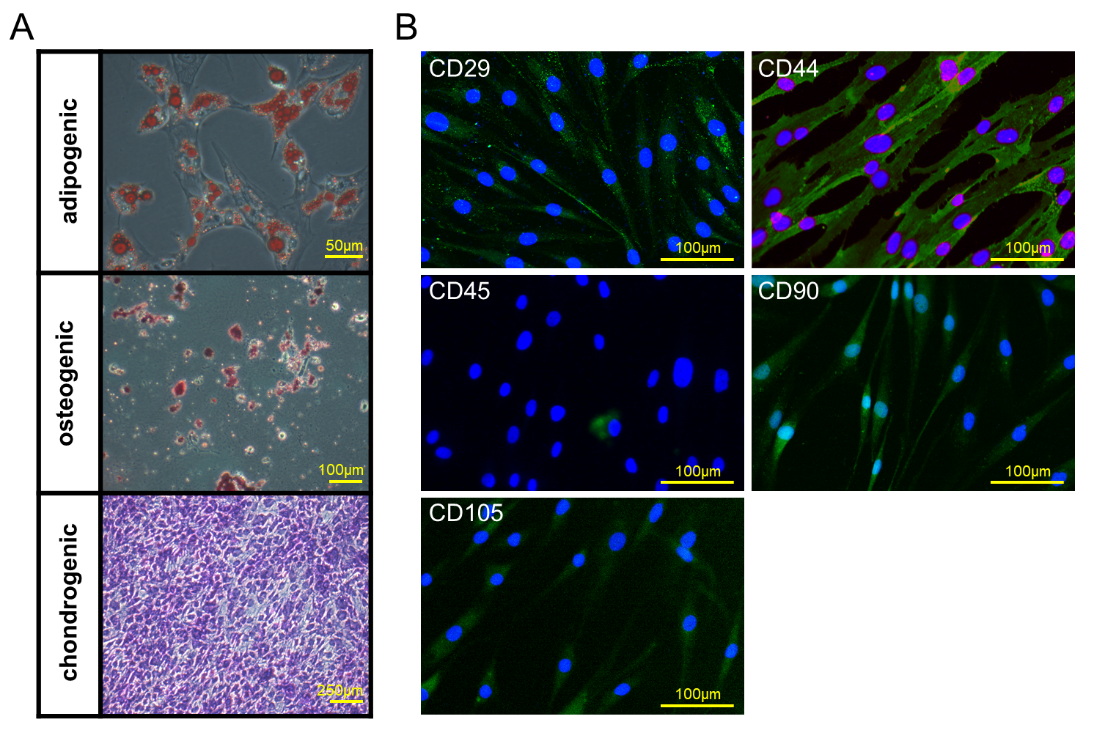


**Figure S1. Characteristics of ADSCs.** (A) Image of ADSCs underwent adipogenic differentiation (oil red O staining), osteogenic differentiation (Alizarin Red staining) and chondrogenic differentiation (Toluidine Blue staining). (B) Immunofluorescence staining of ADSCs (CD29, CD44, CD45, CD90, CD105) demonstrated they were positive for CD29, CD44, CD90, CD105 and negative for CD45.

Western blot raw data
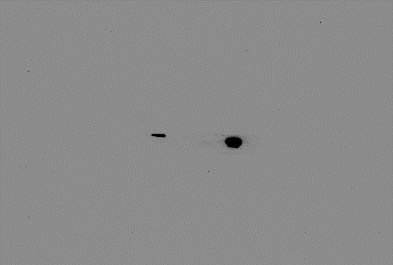

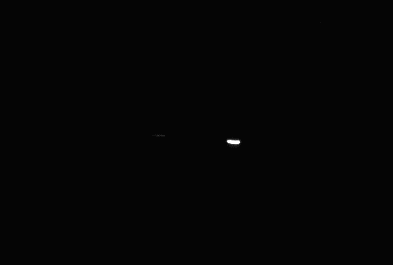


GAPDH (left: ADSC-EV, right: positive control)


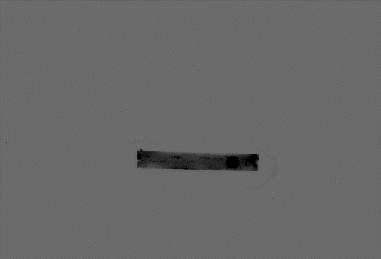

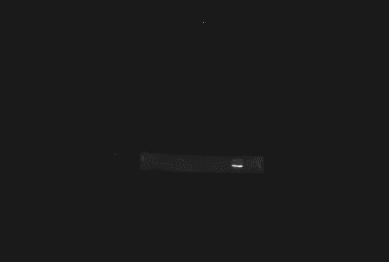


TSG101 (left: ADSC-EV, right: positive control)


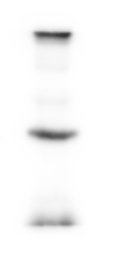

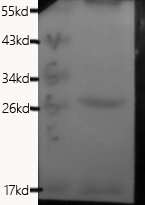


VEGF
